# Exploring the Place of Fish Skin Grafts with Omega-3 in Pediatric Wound Management

**DOI:** 10.3390/jcm13010112

**Published:** 2023-12-25

**Authors:** Ibrahim Cherry, Lana Tarhini, Marie Doan, Anthony De Buys Roessingh

**Affiliations:** 1 Department of Plastic and Reconstructive Surgery, Université Libre de Bruxelles, 1070 Bruxelles, Belgium; 2 Faculty of Pharmaceutical Science, Université Libre de Bruxelles, 1070 Bruxelles, Belgium; lana.tarhini@icloud.com; 3 Pediatric Surgery, Hôpital Riviera Chablais, 1847 Rennaz, Switzerland; marietherese.doan@hopitalrivierachablais.ch; 4 Pediatric Surgery, Centre Hospitalier Universitaire Vaudois, 1005 Lausanne, Switzerland; anthony.debuys-roessingh@chuv.ch

**Keywords:** wound, scar, child, graft, fish skin

## Abstract

Wound healing in the pediatric population is known to be a challenge and poorly studied. Split-thickness skin grafts, full-thickness skin grafts, and flaps overlap their applications with the growing field of cellular and tissue-based therapies. However, their role in pediatric reconstruction has yet to be defined. The Kerecis^®^ Omega-3 wound patch, derived from decellularized codfish skin, has garnered attention due to its preserved microscopic architecture resembling the human extracellular matrix. This acellular dermal matrix acts as a scaffold, fostering dermal cell and capillary adhesion while harnessing omega-3 polyunsaturated fatty acids for granulation acceleration and antimicrobial effects. This study presents a comprehensive review and surgical protocol for utilizing Kerecis^®^ fish skin in pediatric wound care. The research embraces a case series involving five patients with diverse wound locations. The Kerecis^®^ Omega-3 wound patch underwent meticulous application and careful monitoring. The results highlight an average time of 48.6 days for complete epithelialization, yielding favorable outcomes with no hypertrophic scarring and mild retraction. Kerecis^®^ fish skin grafting stands as a tool that not only accelerates healing but also addresses the multifaceted challenges associated with wound management in the pediatric population: the avoidance of donor site morbidity and improved postoperative pain control.

## 1. Introduction

Managing wounds and scars in children presents a significant challenge for surgical and rehabilitation teams. Beyond the aesthetic implications, the wound healing process can be painful, and scarring may disturb child growth leading to restriction of joint range motion, subsequently causing disabilities and loss of quality of life [1]. Current reconstructive methods, such as skin grafts and flaps, overlap with emerging cellular and tissue-based therapies. However, their specific application in pediatric reconstruction remains to be clearly defined [2].

Explorations into acellular dermal matrix preparations as an alternative paradigm have demonstrated promising results in individual studies, emerging as an encouraging adjunct to promoting wound healing outcomes [3,4,5,6]. Recently, there has been a notable focus on decellularized tissues sourced from non-mammalian origins, driven by reduced ethical constraints and a diminished risk of infection transmission (e.g., foot-and-mouth disease, swine influenza, and bovine spongiform encephalopathy) [7]. The avoidance of disease transmission risks and the abundant availability of disposable fish waste, particularly fish skin, have captured the interest of numerous researchers. Consequently, various decellularization techniques have been employed to develop an efficacious product serving as a biological scaffold. The epidermis of fish, similar to the epidermis found in terrestrial vertebrates like mice or humans, comprises a multi-layered tissue distinctly separated from the dermis by a basement membrane [8,9].

The Kerecis^®^ Omega-3 wound patch (Kerecis Ltd., Isafjordur, Iceland) emerges as a novel avenue for consideration. This acellular dermal matrix is derived from decellularized codfish skin. The fish skin needs only mild processing, thereby its microscopic architecture is preserved. Being close to the human extracellular matrix, it acts as a scaffold for the ingrowth and the adhesion of dermal cells and capillaries. Scaffolds, made from synthetic or natural biomaterials, serve as structures designed to replicate the cellular microenvironment, commonly known as the extracellular matrix (ECM). Their purpose is to facilitate the growth, proliferation, and organization of cells [10]. Moreover, Kerecis^®^ fish skin contains Omega-3 polyunsaturated fatty acids with a large concentration of docosahexaenoic acid (DHA) and eicosapentaenoic acid (EPA), which are associated with anti-microbial and anti-inflammatory properties [11,12].

Previous studies have already explored the potential of Kerecis^®^ fish skin in hard-to-heal lower extremity chronic ulcers and deep-burn management [13,14,15,16,17,18,19,20,21,22]. Despite its demonstrated efficacy, the pediatric field in this matter is poorly documented [23,24,25]. To bridge this gap, we undertook a comprehensive literature review and developed a surgical protocol, leading us to initiate a case series. The purpose of this study is to assess the safety and outcome of intact fish skin when used as dermal/skin replacement therapy in the pediatric population.

## 2. Materials and Methods

This study was conducted in adherence to our hospital ethical guidelines (University Hospital of Lausanne). Informed consent was obtained for each patient. Patients included in this study met the following criteria: aged under 18 years old, underwent Kerecis^®^ fish skin grafting following a rigorous protocol (see below) and followed for a minimum period of 6 months. Patients enrolled in this study were identified retrospectively. Their medical records were reviewed, and data was collected concerning patient demographics, initial mode of injury, indication for Kerecis^®^ placement, operative data, postoperative care, complications, and outcomes.

### Kerecis^®^ Omega-3 Wound Patch Placement Protocol

The acellular dermal matrix was obtained from North Atlantic codfish skin (Gadus morhua) farmed in Isafjordur, Iceland. The process of preparing codfish skin for use as an acellular dermal matrix involves several crucial stages. The collected skin undergoes a thorough decellularization process to remove cellular components, minimizing the risk of immune reactions. After meticulous cleaning, the skin is sterilized to eliminate potential pathogens. This process is deliberately gentle to preserve the natural microscopic architecture, which serves as a scaffold for cell growth. Omega-3 polyunsaturated fatty acids, known for their wound-healing benefits, are retained during the process. The prepared matrix is finally packaged and stored under proper conditions to maintain sterility and therapeutic properties [2].

In the placement of Kerecis^®^ acellular dermal matrix, the methodology follows a standardized approach overseen by the senior author to ensure consistent coverage procedure and post-operative care, drawing insights from the available literature. As a preventative measure, an intravenous antibiotic was administered 30 min before surgery. The procedure encompassed a meticulous debridement targeting colonized and non-viable tissue to reveal viable and bleeding tissue. Bleeding is controlled with cauterization and epinephrine-soaked gauze pads. The Kerecis^®^ Omega-3 wound patch is first cut to fit a slightly larger surface than the wound (1 cm away from the wound edge) and is gently rinsed in sterile physiologic saline solution for one minute. This overlap is made to allow for any slight slippage and is due to mild contraction of the graft after salination and insertion in the wound bed. Close contact between the graft and the wound bed is achieved to facilitate cellular ingrowth and integration. The graft is covered with a non-adherent dressing. In the further course, the wound is inspected in our outpatient clinic every 3–5 days depending on clinical indication. The matrix remained in situ until its complete disintegration. Limited local debridement is performed to remove non-viable tissue and support optimal healing conditions. In the assessment of treatment involving acellular dermal matrices from fish skin, vigilant monitoring is essential to promptly identify potential complications. Infection, marked by redness, swelling, and discharge, is closely observed due to its potential to impede healing. Additionally, allergic reactions, such as itching, rash, or breathing difficulties, are also monitored.

## 3. Results

The matrix has been used highly selectively in five patients (Table 1). The consecutive patients were treated in the region of the scalp (1), the axillary region (2), and the lower extremity (2). Our sample consisted of three males and two females with a mean age of 8.6 years old (range from 1.8 to 16 years old). Patients underwent fish skin graft placement between April 2022 and February 2023.

Patient 1 is a 21-month-old boy who sustained a thermal injury on the left foot, covering 2% of his total body surface area (TBSA). Wound healing with repeated dressing changes failed to accomplish epithelialization because of the profound nature of the burns. Notably, split-thickness skin grafting was initially performed, but graft integration did not succeed. Ultimately, wound debridement and Kerecis^®^ fish skin graft were performed and allowed wound contraction within the first 10 days and wound coverage within 29 days.

Patient 2 is a two-year-old boy who experienced a 3rd-degree and deep 2nd-degree thermal burn extending to both his lower limbs and covering a total of 14% of his TBSA. Despite attempted wound healing through repetitive dressing changes, his follow-up was immediately marked by delayed healing in the popliteal fossa. Split-thickness skin grafting did not succeed, certainly due to graft location. Subsequently, debridement and the application of a Kerecis^®^ fish skin graft were pursued, resulting in notable wound contraction within a few days and complete wound coverage achieved within 59 days.

**Figure 1:**
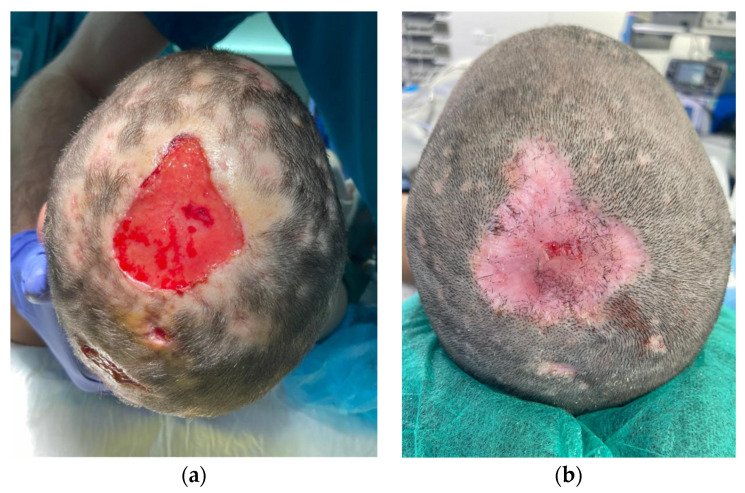
Descriptive picture of patient 3. Two weeks (**a**) and two months (**b**) after Kerecis^®^ placement.

Patient 3 is a 9-year-old girl with a five-by-five cm hairless swollen mass on the vertex, initially diagnosed as an infected hematoma and therefore drained under general anesthesia. The diagnosis was questioned after the persistence of a tissue defect and an unfavorable clinical evolution under antibiotics. When fungal filaments and spores were identified upon culture, a definitive diagnosis of tinea capitis with fungal kerion was made. Clinical improvement was obtained with local and oral anti-fungal therapy. However, a tissue defect on her scalp was left persistent (Figure 1). Successful and painless wound coverage was obtained with a fish skin graft. Complete epithelialization was obtained two months later. A pinwheel rotation flap was later performed for coverage of the alopecic area.

Patient 4 is a 16-year-old boy followed by our reconstructive surgery team for 4 years. He experienced a thermal burn extending to the left shoulder and the axillary region, covering 3% of his TBSA. Upon long-term follow-up, he developed a restrictive scar contracture of the affected area. The scarring process at this previously grafted site led to a notable limitation in active and passive shoulder abduction (50°). Our attitude involved conducting a thorough scar excision and subsequent placement of a Kerecis^®^ fish skin graft. Immediate post-operative monitoring showed no complications. By the 6th month following surgery, the scarring showed no sign of contracture or hypertrophy, and complete restoration of shoulder range of motion was achieved.

Patient 5 is a 14-year-old girl followed by the pediatric surgery team for the management of hidradenitis suppurative lesions on the right axillary region. Local dressing and oral antibiotics showed no significant clinical improvement and the quality of life remained low. Surgical excision of the lesion with adjunct reconstruction using a Kerecis^®^ fish skin graft was proposed. The wound size was 11 × 10 cm. Healing was achieved 41 days later with no scar contracture.

All patients underwent meticulous wound debridement and patch application under general anesthesia and were discharged the same day. The wound surface ranged between 10 and 110 cm^2^ (mean: 53 cm^2^). Standard analgesic medications were taken for a maximum period of 48 h in all patients. Upon follow-up at our outpatient clinic, shrinkage in the wound surface was observed in all patients after a few days, followed by early wound granulation. The mean time required to achieve complete epithelialization was 48.6 days (range: 29–62 days). No hypersensitivity or allergic reaction was reported. Regarding the management of all the scars, all the enrolled patients underwent physiotherapy for scar massage and wore custom-made clothing for pressure therapy. The definitive outcome was satisfactory in all wounds, meaning complete wound coverage and absence of scar contracture.

## 4. Discussion

As this is a case series, we cannot make claims of effectiveness which would require a more rigorous clinical trial. However, the findings reveal that the Kerecis^®^ fish skin graft with Omega-3 is an adequate and safe scaffold in the process of repairing damaged tissues in the pediatric population. A quality skin replacement was accomplished in all patients, with no donor site morbidity.

The efficacy and safety of fish skin grafting have been widely demonstrated in the adult population. Notably, two different randomized controlled clinical trials have shown the superiority of this product over human amnion/chorion membrane [26] and porcine small intestine submucosa matrix, when used in full-thickness wounds [27]. Mammalian scaffolds require rough processing to reduce viral and prion transmission risk; such risk is non-existent in cod fish skin. Therefore, its microstructure and composition are preserved thanks to softer processing [18,26]. Magnusson et al. showed a significantly larger three-dimensional cell proliferation when using the acellular fish skin matrix [26]. Additionally, when compared to conventional dressing such as silver or alginate dressing, fish skin grafts seem to be superior in terms of time until epithelialization [28]. While the exact role of fish skin in accelerated wound healing requires further study, the following features have been described throughout the literature: prevention of bacterial contamination and hydroelectrolytic losses, stimulation of epithelization, and acceleration of wound granulation [29].

When addressing wound healing in children, obtaining rapid epithelialization is crucial for minimizing the risk of infections, but as importantly, reducing pain and addressing psychological concerns. A positive effect of omega-3 fatty acids on nociception has been previously observed, both in animal models [30] and in a case series [31]. This finding is supported by our observation since all patients could be discharged with minimal analgesic use, and further treatment carried out in the outpatient setting. Additionally, the matrix of fish skin undergoes gradual disintegration over the wound surface, without the need for removal, thus limiting pain during care and repetitive trauma to the wound bed. Furthermore, the absence of a donor site simplifies the procedure and eliminates associated morbidities such as additional scar formation, pain, and the risk of infection [32].

As illustrated in this report, the clinical application of fish skin grafts expands to chronic wound management and scar reconstruction. Omega-3 fish skin grafts create a conducive environment for chronic wounds, where the healing process is compromised due to factors such as inflammation, colonization, infection, impaired blood supply, and an inability to transition to the proliferative and remodeling phases. For scar reconstruction, the integration of Omega-3 fish skin grafts could provide multiple benefits. They act as scaffolds to promote cellular infiltration and tissue remodeling, potentially reducing scar hypertrophy and enhancing the cosmetic appearance of scars.

The process of wound healing in the pediatric population is known to be unique [22,33]. Children have limited availability of donor tissue and are more susceptible to hypertrophic scarring. More concern about scar retraction is raised in children since it leads to growth disturbances and functional impairment [33]. As previously mentioned, various reconstructive strategies are available to achieve wound closure, all of which have their individual advantages and disadvantages. Upon the application of fish skin grafts, wound contraction was evident in all patients during the initial 14-day period. This observation may suggest the stimulation of myofibroblast activity and is particularly useful in the pediatric population given their inherent greater skin elasticity.

Fish skin grafting has been commonly described as a one-stage procedure to promote second-intent wound healing [16,27,28,29,32], but can also be imagined as a two-stage procedure with a subsequent autograft. Even though the healing process is prolonged in the one-stage procedure, it was still the strategy used in all patients. This predilection for an extended healing period was deliberated and selected in favor of the potential sequelae of additional scarring and the concomitant pain arising from donor site morbidity.

While the obtained results are promising, future considerations regarding the utilization of this matrix should consider its comparatively elevated cost when relative to other dressings. To mitigate the financial aspect, a plausible strategy could involve the sequential application of acellular fish skin matrices at distinct stages of the treatment continuum. For instance, deploying these matrices immediately post-surgery and during the granulation phase could potentially optimize the healing process while concurrently addressing cost concerns.

Globally, this report supports three statements when using Kerecis^®^ fish skin graft as a reconstructive one-stage procedure in the pediatric population: the avoidance of donor site morbidity, improved postoperative pain control, and qualitative skin replacement.

Limitations: The study involves a small sample size of only five patients. A larger and more diverse sample would strengthen the generalizability and reliability of the findings. The study is retrospective, relying on the review of medical records. This design may introduce bias, and prospective studies with standardized protocols and controls would enhance the validity of the results. The study relies on subjective outcome measures such as pain reports. Objective measures and standardized tools for pain assessment could enhance the robustness of the results.

## 5. Conclusions

The utilization of the Kerecis^®^ fish skin graft with Omega-3 as a reconstructive tool in the pediatric population yields substantial benefits. Through its capacity to preserve skin elasticity, stimulate myofibroblast activity, and exploit the analgesic potential of Omega-3 fatty acids, Kerecis^®^ fish skin emerges as a promising avenue for optimized wound healing. The presented cases, reflecting diverse wound types and anatomical sites, further strengthen the foundation for adopting this approach across pediatric reconstructive procedures. As the understanding of pediatric wound healing nuances expands, Kerecis^®^ fish skin grafting stands as a tool that not only accelerates healing but also addresses the multifaceted challenges associated with wound management in the pediatric population.

## Figures and Tables

**Table 1:** Detailed demographical and clinical data.

Sex, Age	Indication	Site (Surface)	Previous Surgery	Epithelialization	Follow-Up	Outcome
M, 1.8 y	3rd degree burn	L. foot (10 cm^2^)	Debridement and STSG	29 days	10 months	Satisfactory
M, 2 y	3rd degree burn	R. knee (25 cm^2^)	Debridement and STSG	59 days	10 months	Satisfactory
F, 9 y	Chronic wound	Scalp (20 cm^2^)	Fungal kerion excision	62 days	18 months	Satisfactory
M, 16 y	Scar revision	L. axillary region (100 cm^2^)	Steroids injections	52 days	10 months	Satisfactory
F, 14 y	Hidradenitis suppurative	R. axillary region (110 cm^2^)	Excision of HS lesion	41 days	12 months	Satisfactory

HS: hidradenitis suppurative. STSG: split-thickness skin graft. Satisfactory: no retraction, no hypertrophic scar and complete wound coverage.

## Data Availability

The data presented in this study are available on request from the corresponding author. The data are not publicly available due to to privacy and ethical considerations, as well as compliance with institutional review board regulations.
